# Exploring the Diets of Adults with Obesity and Type II Diabetes from Nine Diverse Countries: Dietary Intakes, Patterns, and Quality

**DOI:** 10.3390/nu12072027

**Published:** 2020-07-08

**Authors:** Jade Willey, Marian Wakefield, Heidi J. Silver

**Affiliations:** Vanderbilt University Medical Center, 1211 Medical Center Dr, Nashville, TN 37232, USA; jade.willey@vumc.org (J.W.); marian.wakefield@vumc.org (M.W.)

**Keywords:** dietary intake, diet quality, dietary patterns, diabetes, obesity

## Abstract

Background: Calorie-dense diet is a main driver of the global epidemics of obesity and type 2 diabetes (T2DM). While various dietary strategies and patterns are efficacious in reducing risk and improving glycemic control, dietary intake and diet quality have been inadequately studied among individuals who remain living in their native environments. There is also little published on dietary patterns of diverse ethnic, cultural, or regional populations. Objective: To explore dietary intakes, patterns and overall diet quality in adults with obesity and T2DM from diverse countries. We hypothesized that individuals sharing a common clinical phenotype (age, BMI, years since T2DM diagnosis and inadequate glycemic control) would demonstrate comparable high calorie “western” dietary patterns and low diet quality despite differences in geographic regions and cultures. Design: Diet data were acquired from 611 adults in Argentina, Germany, Poland, Serbia, Slovakia, Slovenia, Spain, Turkey and the USA via three 24-h diet recalls. Contribution of 168 foods to 14 primary food groups was confirmed by Spearman’s rank-order correlations and Principle Component Factor Analysis identified dietary patterns. Diet quality was assessed using the Healthy Eating Index 2015. Results: Eleven dietary patterns were extracted; the most common were a “Mediterranean-like” pattern shared by six countries and a “Calorie Dense” pattern shared by five countries. Also common were “Lacto-Vegetarian, “Pesco-Vegetarian,” and “Vegan” patterns. Only 2.1% of subjects had good diet quality (HEI-2015 score >80). Conclusions: The diet pattern data suggest that influences of more traditional region-specific diets remain. However, overall diet quality was poor and may contribute to inadequate glycemic control, possibly due to excess intake of high calorie/nutrient poor foods, which may be associated with global transitions occurring in the available food supply.

## 1. Introduction

Despite the World Health Organization’s goal to reduce the prevalence of type 2 diabetes mellitus (T2DM), the incidence of T2DM has escalated rapidly from a local public health concern to a global pandemic now affecting over 415 million individuals worldwide. The health and economic burden of T2DM, estimated to reach a healthcare cost of $2.1 trillion by 2030, derives primarily from its macro- and microvascular complications resulting from chronic poor glycemic control [1,2]. Although genetics, ancestry, family history, socioeconomic status, and older age are contributing factors, the main drivers are environmental: consuming a calorically dense diet, sedentary lifestyle, and the development of excess adiposity (obesity) [3].

Prior strong evidence from randomized controlled trials and prospective observational studies, including findings from the long-standing Diabetes Prevention Program [4], has shown that reducing energy intake (and engaging in moderate physical activity) can achieve weight loss and glycemic control, as well as reduce or delay the incidence of T2DM in high-risk individuals. Consequently, multiple organizations worldwide, including the World Health Organization, the United Nations Food and Agriculture Organization, the International Diabetes Federation, and the American Diabetes Association, have clinical guidelines that emphasize following a more healthful eating pattern and consuming a more nutrient dense diet [5,6,7,8].

However, increasing internationalization of food systems over the past few decades has resulted in widespread availability and affordability of “westernized” food and beverage items [9]. The increase in the proportion of highly processed foods and sugars in the food supply worldwide, replacing traditional diets based on indigenous customs and locally grown foods, may adversely impact the overall nutritional composition of habitual intakes. Although a study using data from 70 high- and middle-income countries to estimate the social and economic effects of the globalization of the food supply showed no significant relationship with the overall nutritional composition of the diet or the prevalence of T2DM [10], an ecological analysis using food product availability databases from 96 countries showed the degree of exposure to a “westernized” diet had a dose–response relationship with the prevalence of T2DM [11].

While it is understood that ethnic and cultural traditions influence food preferences and choices, it remains unclear how much influence the global accessibility of calorie dense westernized foods has had on key characteristics of the diets of people who remain living in their native environments and have both obesity and T2DM. The purpose of this study was to explore the dietary intakes of individuals living in several distinct countries who share a common clinical phenotype characterized by similar age, high BMI, years since diagnosis of T2DM, and inadequate glycemic control. To do so, we performed diet assessments that would identify dietary nutrient composition, dietary patterns, and overall diet quality. We hypothesized that dietary intakes would be of poor dietary quality and would reflect a “western” dietary pattern characterized by high intake of red and processed meats, dairy products, refined grains, and sugar sweetened beverages with minimal intake of fruits, vegetables, legumes, and whole grains. While global recommendations exist, most of the countries in this study do not have country or region-specific guidelines for management of T2DM, thus the combination of diet analysis methods employed provides novel information to assist with the development of guidelines derived from greater understanding of current local daily food consumption.

## 2. Materials and Methods

### 2.1. Subjects

The present findings derive from analysis of baseline dietary data from adults with T2DM who agreed to participate in a randomized clinical trial comparing diet versus insulin (detemir) intervention for glycemic control [12]. Subjects were recruited by flyers posted at 110 outpatient clinic sites across nine countries that have over 6% of their adult population diagnosed with T2DM (Argentina, Germany, Poland, Serbia, Slovakia, Slovenia, Spain, Turkey, and the United States). Subjects were enrolled if they were age ≥21 years, overweight/obese (BMI 25–45 kg/m^2^), diagnosed with T2DM at least 6 months prior, on oral anti-diabetic medications (metformin alone or combined with another oral anti-hypoglycemic medication) with dosage unchanged for at least 3 months prior, were insulin-naïve, and had glycated hemoglobin (HbA1c) between 7.0–9.0% indicating inadequate glucose control. Potential subjects were excluded if they had diagnosed cancer, heart, liver or kidney disease; recurrent severe hypoglycemia; medications that affect appetite or weight; food allergies, avoidances or other dietary restrictions; or were pregnant or breastfeeding. Institutional Review Board approval was obtained at each of the sites, including the Ethics Committee of the Vanderbilt University Medical Center Institutional Review Board (IRB#081051), and all subjects provided written informed consent for their participation.

### 2.2. Diet Assessment

**Figure 1:**
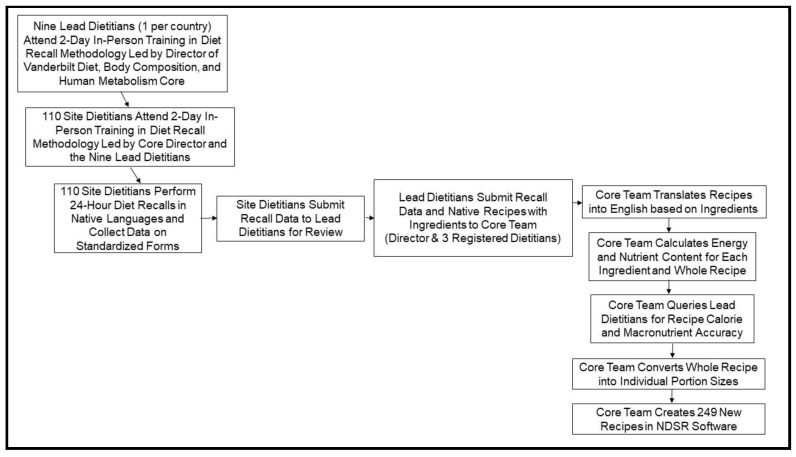
Process of study diet data collection of 1833 24-hr recalls from 9 countries and diet data entry by Vanderbilt Core team into Nutrition Data System for Research (NDSR) database.

Dietary intakes were assessed during a 12-week period using the U.S. Department of Agriculture (USDA) five-step multi-pass 24-h diet recall methodology [13,14,15,16]. Prior to data collection, diet recall method was standardized across the nine countries by training of an extensive dietitian network led by the Vanderbilt Diet, Body Composition and Human Metabolism Core (Core). Two in-depth weekend-long in-person training workshops were convened; first with the nine lead dietitians who were selected to represent each participating country and then with the 110 site dietitians who were trained to administer diet recalls directly to all subjects within their study site in their native languages (Figure 1). Prior to initiating diet recall interviews, the site dietitians familiarized enrolled subjects with estimating portion sizes using standard measuring utensils (plates, cups, bowls and spoons of various sizes). Site dietitians then acquired diet recall data by phone on two weekdays and one weekend day that were unannounced, randomly selected, and nonconsecutive. Site dietitians transmitted all diet recall data electronically to their respective country’s lead dietitian who was responsible for assuring uniform data collection procedures, completeness of recall details, and identifying region-specific or homemade food items that would likely be unfamiliar to the Core dietitians. The lead dietitians provided recipes with lists of ingredients for the region-specific and homemade food items to the Core dietitians who converted these 249 recipes into English language recipes based on the individual food ingredients, energy and macronutrient contents, and portion sizes using the USDA National Nutrient Database for Standard Reference [17]. All recipe ingredient energy and nutrient data were confirmed with the nine lead dietitians prior to entry into the Core’s Nutrition Data System for Research software database (NDSR version 2014, Nutrition Coordination Center, University of Minnesota). This process allowed inclusion of all food and beverage items from all 1833 24-hr diet recalls, including the regional/ethnic/cultural–specific items, into NDSR, NDSR output files, and the statistical analyses.

### 2.3. Diet Data Analysis

First, diet energy and nutrient composition was determined based on the NDSR intake properties totals file which provides the average daily intake for 165 nutrients, nutrient ratios/indices, and other food components. Second, the NDSR serving count totals file was used to explore dietary patterns from calculating the average daily intake of 168 food and beverage subgroups. The 168 food and beverage subgroups were combined into 14 primary food groups based on similar component ingredients as well as the energy and macronutrient composition within a standard serving (Supplementary Materials Table S1). Cumulative servings of items in each of the 14 primary food groups were calculated for each subject and entered into a Microsoft Excel spreadsheet prior to dietary pattern analysis.

Three NDSR output files (the serving count food file, the intake properties totals file, and the component/ingredient file) were used to determine diet quality based on scores from the Healthy Eating Index-2015 (HEI-2015), a tool created by the U.S. Department of Agriculture and the National Cancer Institute to evaluate adherence to the *Dietary Guidelines for Americans* [18]. The HEI-2015 tool provides scores for 9 adequacy variables (total fruits including 100% fruit juice; whole fruits, total vegetables including legumes, greens and beans; whole grains; total dairy; total protein foods; seafood and plant proteins; and the ratio of unsaturated to saturated fatty acids) and 4 moderation variables (refined grains, sodium, added sugars and saturated fats) and an overall diet quality score that ranges from 0–100 [19]. A score <51 was considered poor diet quality and ≥80 as high overall consistency with the *Dietary Guidelines* [20]. Prior to calculating each subject’s scores for these components via the simple ratio method, the University of Minnesota Nutrition Coordination Center methodology was followed to generate revised variables that conformed with the units of measure used in the HEI-2015 (number of servings in cup or ounce equivalents per 1000 kcal) [20].

### 2.4. Statistical Analysis

Statistical analyses were performed using SPSS (version 23, IBM, Armonk, NY, USA). Demographics and nutrient intakes were summarized using means and standard deviations for continuous variables and percentages for categorical variables. Principal component factor analysis (PCA) was used to identify dietary patterns since it is a commonly used empirically driven method that enabled summarizing the patterns of the habitual dietary intakes of subjects within each country. Importantly, PCA determines linear intercorrelations between food and beverage items while overcoming collinearity confounding [21]. It is understood that robust correlations between the 168 food items and the 14 primary food groups are necessary for PCA to have value. Thus, a correlation matrix was generated for each country to examine the relationships between the individual food items and the primary food groups (Supplementary Materials Figure S1). These matrices showed positive correlation coefficients that indicated using PCA would be an appropriate method to identify dietary patterns using the food items selected. Since dietary patterns may be population or region-specific, we did not attempt to fit a priori defined patterns into the statistical modeling. The PCA was performed with Varimax orthogonal rotation to simplify the structure and improve interpretability [22]. Dietary patterns were generated based on the factors with the highest correlation coefficients for the 14 food groups. The number of factors to retain was determined first by having an eigenvalue >1.0 (a value indicating significant contribution to the variance in food consumption explained by the components) and then examination of scree plots of the eigenvalues to identify the number of dietary patterns that covered at least 40% of the cumulative variance in average dietary intakes within a country. For each of the nine countries, a total of 3 or 4 factors provided a sufficient proportion of the cumulative variance and was the most parsimonious. Primary food groups with positive PCA loadings at r ≥ 0.20 indicated a direct relationship with a particular factor. Within each country, the factors were assigned a dietary pattern label (e.g., “western”) based on the food groups that had the highest positive correlation coefficients within a factor because higher coefficients indicate that the food group contributed more to the derivation of the factor. For diet quality analysis, HEI-2015 component and total scores were summarized using means and standard errors. One-way analysis of variance (ANOVA) was used to assess differences in mean intakes and HEI-2015 scores among the nine countries.

## 3. Results

### 3.1. Demographics

A total of 1132 individuals were screened and 611 were enrolled and completed three diet recalls at baseline for a total of 1833 24-hr recalls. No significant differences were observed at baseline by country in demographics or diabetes status.^12^ Subjects’ average age was 57.4 ± 9.8 years and average BMI was 34.4 ± 5.5 kg/m^2^. Almost half of subjects were female (48.7%), 89.4% were Caucasian, 7.8% were African American, and 23.3% were of Hispanic ethnicity. The average time since diagnosis of T2DM was 8.6 ± 5.9 years and average HbA1c was 8.0 ± 0.7%. About one third (36.3%) were taking Metformin alone, and the rest Metformin in combination with another oral anti-hyperglycemic medication. All subjects had a low physical activity level based on their International Physical Activity Questionnaire (IPAQ) scores [23,24].

### 3.2. Dietary Intakes

Reported energy intakes ranged from 1611.4 ± 431.9 kcal/day in Slovakian subjects to 2271.7 ± 1154.9 kcal/day in German subjects, with an overall average of 2004.2 ± 878.3 kcal/day (Table 1). Caloric density (kcal/g) of dietary intakes differed across the nine countries (F = 3.2, *p* = 0.001), with the most calorie dense diets in Spanish and German subjects. Although percentage of energy intakes as total carbohydrate did not differ across the nine countries (F = 1.5, *p* = 0.16), there were significant differences in the consumption of total sugars, added sugars, and overall glycemic load (*P*s < 0.001). The highest consumption of sugars was in subjects from Spain, USA, and Turkey and the lowest consumption of sugars in subjects from Poland, Serbia, and Slovakia. In Spanish and Turkish subjects, high total sugars intake was driven by high fructose intake whereas American subjects derived more total sugars from high sucrose intake.

There was no significant difference in the percentage of fat from saturated fat (SFA) across the nine countries (F = 1.6, *p* = 0.12), but the percentage of fat from mono- (MUFA) and polyunsaturated (PUFA) fats differed (*P*s = 0.001) with the highest percentage of MUFA consumed by Spanish and Turkish subjects and the highest percentage of PUFA in Slovenian subjects. Overall, the ratio of unsaturated to saturated fats differed across the nine countries (F = 3.8, *p* < 0.001) with Spanish subjects consuming the highest proportion of unsaturated fats and German subjects the least. The nutrients that differed the least across the nine countries were the total amount of protein consumed (*p* = 0.84) and sodium intake (*p* = 0.05). Overall, sodium intakes were high, ranging from 3115.7 ± 1703.1 mg/d in Argentine subjects to 4509.4 ± 2693.2 mg/d in Turkish subjects (Table 1).

### 3.3. Dietary Patterns

Extracting three to four components overall from PCA explained 42–70% of the total variation in dietary intakes (42% in American, 56.5% in Argentinian, 48.2% in German, 51.7% in Polish, 57.6% in Serbian, 60.9% in Slovakian, 69.6% in Slovenian, 59.6% in Spanish, and 56.3% in Turkish intakes). The scores that were ≥0.20 for each of the factors derived for each country are presented in Table 2. From these factors, there were eleven different dietary patterns identified (Table 3): a breakfast pattern (cereals, breads, grains, dairy, fats); a bland pattern (white meats, fish, dairy, saturated and polyunsaturated fats); a low carbohydrate pattern (fruits, vegetables, red and white meats); a high fat pattern (red meats, saturated and polyunsaturated fats); a calorie dense pattern (grains, starches, desserts, sweets, fats); a vegetarian-lacto pattern (fruits, vegetables, legumes, dairy, desserts, sweets, fats, water); a vegetarian-pesco pattern (fruits, vegetables, legumes, fish, desserts, sweets, fats); a vegan pattern (fruits, vegetables, legumes, water); a mediterranean-like pattern (fruits, grains, dairy, white meats, fish, monounsaturated fats); a varied pattern (vegetables, breads, starches, grains, legumes, red meats, dairy, fats and water); and a hearty pattern (vegetables, breads, grains, starches, red meats, dairy).

The Mediterranean-like pattern extracted was the most common, shared by subjects from six countries (Argentina, Poland, Serbia, Slovakia, Slovenia, and Spain). The foods most frequently consumed across these countries and contributing most to the variance in the Mediterranean pattern were oranges, apples, whole grain breads, lean white fish such as cod, trout and carp, and various cheeses (Table 3). The next most common pattern was the “calorie dense” pattern with subjects from Germany, Slovakia, Spain, Turkey, and the U.S.A. sharing this pattern. The foods most frequently consumed that contributed most to the variance in the “calorie dense” pattern and were common among these countries were homemade or bakery breads, rice and rice pilaf, local pastries, homemade and bakery cakes, and full-fat cheeses including Gouda, goat, and cheddar. Also common were the “vegetarian-lacto” pattern shared by subjects from Argentina, Poland, Serbia and Slovenia, and the “vegan” pattern shared by subjects from Germany, Serbia, Slovenia and Turkey. Frequently consumed foods that contributed most to the variance in the vegetarian-lacto and vegan patterns across the countries were apples, bananas, oranges, tangerines, beans, and bean soups. The least common pattern appeared in only one country, a “bland” pattern, which was comprised of breaded and fried white meats (chicken and pork), white fish, and dairy items that accounted for 13.4% of the variance in the dietary intakes of Slovakian subjects.

### 3.4. Dietary Quality

Overall diet quality was poor with HEI-2015 total scores averaging 52.89 ± 0.55 and ranging from the lowest average score of 47.93 ± 0.31 in German subjects to the highest average score of 63.78 ± 0.65 in Slovenian subjects (Table 4). The countries with the greatest proportion of subjects in the poor diet quality range (total HEI score <51) were Germany (62.2%), Poland (43.2%), Argentina (42.6%), United States (42.5%), and Serbia (41.7%). In contrast, three countries had less than 1/3 of subjects with poor diet quality (Slovenia: 11.8% of subjects, Slovakia: 26.7% of subjects, Turkey: 27.6% of subjects) (Table 5). The individual component scores most strongly associated with lower total HEI scores were refined grains and empty calories (r = 0.55 and r = 0.48, respectively, *P*s < 0.001). Higher total HEI scores correlated most strongly with whole grain and total fruit scores (r = 0.48 and r = 0.47, respectively, *P*s < 0.001). While average whole grain scores did not differ across the nine countries (*p* = 0.14), total fruit scores were significantly different (*p* < 0.001) showing low scores of 1.3 ± 0.1 servings per day in German subjects and 1.7 ± 0.1 servings per day in United States subjects. Spanish subjects had the highest scores for total and whole fruits (3.0 ± 0.4 and 3.7 ± 0.4 servings per day, respectively).

## 4. Discussion

The number of adults living with T2DM continues to rise rapidly, although there are some differences in prevalence by geographical region. For example, the countries included in the present study have a 2017-estimated prevalence of adults diagnosed with T2DM ranging from 6.2% of Argentinian to 14.3% of Serbian adults [25]. While the etiology of T2DM is complex, a “western” dietary pattern has been implicated in this epidemic [26]. In contrast, a higher quality diet characterized by consumption of more fruits, vegetables, whole grains, white meats and fish has been associated with reduced risk for T2DM [27]. Based on the lack of glucose control evidenced by the HbA1C levels of the study subjects, we hypothesized that a “western” pattern would be most common across the nine countries despite the differences in geographic regions and local food environments.

Contrary to our hypothesis, the most novel finding of this study was that a “Mediterranean-like” dietary pattern characterized by the consumption of fruits, vegetables, whole grains, white meats, fish, and monounsaturated fats was the most common pattern among the nine countries. Notably, four recent meta-analyses showed that consuming a Mediterranean diet is associated with a 0.3 to 0.47% reduction in HbA1c [28,29,30,31]. Indeed, when a traditional Mediterranean diet was provided to Australian adults with T2DM their HbA1c reduced from 7.1% to 6.8% in 12 weeks [32]. Further, prospective studies have shown that a “Mediterranean” dietary pattern reduces risk for oxidative stress, inflammation, and the microvascular complications of having T2DM [33,34]. In the present study, the “Mediterranean-like” dietary pattern was identified in subjects from countries on or near the Mediterranean Sea coastline—Slovenia, Serbia and Spain. However, this is just one factor in the adoption of a Mediterranean type diet. It is noteworthy that this pattern also emerged in Slovakian, Argentinian and American subjects, suggesting that diabetics may be adopting this pattern after diagnosis as a means of improving their health status. Indeed, local adaptations of a Mediterranean-style pattern have been emerging in non-Mediterranean populations as this diet has attracted much global media attention due to the growing body of evidence revealing a positive association with reducing chronic disease comorbidities [35].

Less surprising, given the large number of subjects with low diet quality, was the next most common dietary pattern—a “calorie dense” pattern characterized by high intakes of refined grains, starches, desserts, sweets, red meats, alcohol, and saturated fats. Although three of the five countries sharing this pattern (USA, Turkey, Germany) are known to have high calorie intakes, all five countries are evidence of globalization of the food supply at the country level, with greater availability of items containing animal-based proteins and added sugars. Consumption of an “calorie dense” pattern has been associated with higher HbA1c and fasting glucose levels [36]. In American adults, a “calorie dense” diet was positively associated with fasting insulin levels and insulin resistance based on homeostasis model of insulin resistance (HOMA-IR) scores [37,38]. Moreover, in the EPIC-Norfolk study of ~22,000 adults, the positive association between calorically dense dietary intake and new-onset T2DM was independent of obesity (BMI ≥ 30 kg/m^2^) [39]. However, the EPIC-Interact study of 15,434 adults showed no significant association between dietary calories (per gram of food) and incidence of T2DM [40]. It is noteworthy that calculation of caloric density in the EPIC-Interact study included solid foods only, excluding sugar-sweetened beverages which can influence total caloric intake and glycemic control.

Another novel finding was the identification of three types of vegetarian patterns from PCA, which we termed “lacto-vegetarian,” “pesco-vegetarian,” and “vegan.” However, the limited evidence on the role of vegetarian and vegan dietary patterns in glucose control as well as the development of T2DM remains inconclusive. One small study in Korean adults showed a 0.3% greater reduction in HbA1c after 12 weeks of vegan compared to conventional diabetic diet [41]. Interestingly, a large study in Canada and the U.S.A. showed lacto-ovo vegetarians, semi-vegetarians, and vegans had lower risk for developing T2DM than nonvegetarians [42]. However, a multinational consortium of prospective studies that included participants aged ≥ 50 years from Greece, the Netherlands, Sweden, Spain, and the U.S.A. showed no association between vegetable and fruit intakes and incident T2DM [43]. It is possible that the influence of assorted types of plant-based foods differ as results combining data from the U.S. Nurses Health and the Health Professionals studies showed that it was plant foods of higher quality that were associated with reduced T2DM risk [44]. As with the “Mediterranean” pattern identified, it is plausible that persons diagnosed with T2DM are attempting to achieve better glucose management by taking advantage of the multitude of dietary approaches now available.

Some limitations of the present study must be addressed. First, these are not nationally representative samples and the number of individuals volunteering to participate at each site varied despite in-depth training in recruitment methods and translation of recruitment materials into native languages. Thus, the findings are not widely generalizable, but rather more exploratory. Second, the food groups, the cut-points used to determine the number of factors to retain in PCA, and the labelling of the factors was somewhat subjective. However, many prior studies have published similar methods to make these determinations. Third, although the methodology used for diet assessment has been well-validated, all diet assessment methods contain some degree of measurement error due to under- and/or over-reporting of intakes. The potential for measurement error was addressed by in-person training of subjects on portion size estimation and by adjusting portions to energy (caloric) intakes. Strengths of the study include the diversity of subjects living in various countries, the extensive network and standardized training of dietitians for diet assessment, capturing dietary data in subjects’ native languages, obtaining recipes and ingredients for homemade or region-specific items from each country so that all foods and beverages consumed were included in data entry and analyses, and the approach for assessment of dietary intake by exploring nutrient intakes, dietary patterns (that were not determined a priori), and overall diet quality.

## 5. Conclusions

The investigation of dietary patterns and diet quality in various ethnicities, cultures, or geographic regions is sparse. Yet, ethnic and cultural dietary traditions influence food preferences and choices. We recognize the dietary patterns generated do not reflect all possible patterns in the populations of these countries since they represent less than 100% of the variability in subjects’ intakes. However, the present findings reflect the overall nutrient composition and dietary quality of foods consumed by adults who share the diagnoses of obesity and T2DM but live in nine distinct countries. Thus, the present data provide some unique insights into the characteristics of dietary intakes in a large sample of individuals who, based on sharing a common clinical phenotype, would be expected to self-manage their glycemia through diet. The present data suggest that while influences of more traditional region-specific diets remain, efforts for glycemic control may be adversely affected by excess intake of high calorie/nutrient poor foods, which may be related to global transitions occurring in the food supply that has increased availability of non-locally grown or non-traditional food and beverage items. Understanding the distinct characteristics of dietary intakes of individuals with T2DM can be used to develop region-specific recommendations and guidelines to improve public health and facilitate changes in clinical practices to help diabetics improve their glucose control. 

## Figures and Tables

**Table 1:** Energy and Nutrient Intakes by Country.

	Argentina	Germany	Poland	Serbia	Slovakia	Slovenia	Spain	Turkey	USA	*p* Value ^a^
Total Amount (g)	2356.3 ± 99.2	2821.9 ± 152.7	2599.2 ± 110.9	2565.2 ± 133.3	2634.6 ± 100.6	2647.9 ± 260.6	2726.0 ± 176.6	3647.3 ± 250.3	2894.1 ± 64.3	<0.001
Energy (kcal)	1768.3 ± 89.8	2271.7 ± 189.9	1708.1 ± 89.8	1882.9 ± 122.7	1611.4 ± 78.9	1967.5 ± 235.9	2245.3 ± 228.0	1952.7 ± 163.6	2115.4 ± 54.9	0.001
Protein (%kcal)	20.5 ± 0.9	18.4 ± 1.1	20.5 ± 0.6	19.9 ± 1.1	19.9 ± 0.8	18.4 ± 1.0	16.6 ± 1.0	18.3 ± 1.1	17.7 ± 0.3	0.001
Fat (%kcal)	34.5 ± 1.1	39.8 ± 1.5	34.4 ± 1.2	36.2 ± 2.1	37.5 ± 1.7	32.9 ± 2.1	38.2 ± 2.7	32.9 ± 1.8	38.1 ± 0.6	0.009
SFA (%total fat)	35.1 ± 1.2	35.0 ± 1.7	33.6 ± 1.1	34.3 ± 1.4	34.8 ± 1.4	30.4 ± 1.6	29.4 ± 1.9	33.4 ± 1.5	33.6 ± 0.5	0.12
MUFA (%total fat)	35.8 ± 0.8	34.3 ± 1.7	35.4 ± 0.8	34.0 ± 0.8	36.2 ± 0.9	36.0 ± 1.4	43.7 ± 1.8	39.4 ± 1.8	36.9 ± 0.3	0.002
PUFA (%total fat)	20.0 ± 1.3	14.9 ± 1.4	18.5 ± 1.1	20.8 ± 1.7	19.7 ± 1.6	24.6 ± 2.4	19.9 ± 1.8	19.4 ± 1.3	21.2 ± 0.5	<0.001
Unsat:SFA (ratio)	0.7 ± 0.1	0.5 ± 0.1	0.6 ± 0.1	0.7 ± 0.9	0.6 ± 0.1	0.9 ± 0.1	0.8 ± 0.1	0.6 ± 0.1	0.7 ± 0.0	0.05
Trans Fat (g)	3.4 ± 0.2	4.1 ± 0.6	2.4 ± 0.3	2.7 ± 0.3	2.2 ± 0.2	2.9 ± 0.6	3.0 ± 0.4	3.6 ± 0.6	3.9 ± 0.2	0.008
Carbohydrate (%kcal)	44.6 ± 1.7	40.3 ± 1.9	44.9 ± 1.4	43.1 ± 2.2	40.7 ± 1.9	45.6 ± 2.4	43.2 ± 2.9	48.8 ± 2.0	43.8 ± 0.7	0.16
Available CHO (g)	182.2 ± 11.0	194.8 ± 21.8	163.6 ± 9.8	169.9 ± 10.7	147.5 ± 9.0	192.5 ± 21.5	210.5 ± 21.5	209.9 ± 16.0	210.9 ± 5.9	0.001
Starch (g)	94.2 ± 7.6	107.3 ± 14.8	96.8 ± 6.2	100.8 ± 7.7	81.0 ± 5.2	111.6 ± 13.6	104.1 ± 12.3	108.0 ± 9.5	113.3 ± 3.7	0.10
Total Sugars (g)	74.7 ± 5.6	74.5 ± 8.7	56.4 ± 4.5	56.2 ± 5.5	53.8 ± 4.5	64.7 ± 8.6	89.6 ± 12.6	84.1 ± 8.3	84.9 ± 3.5	<0.001
Added Sugars (g)	32.1 ± 4.5	47.0 ± 7.7	17.6 ± 2.3	15.7 ± 2.2	23.8 ± 3.1	19.2 ± 4.6	39.4 ± 8.8	29.2 ± 5.0	52.9 ± 3.3	<0.001
Sucrose (g)	23.4 ± 2.6	39.6 ± 7.2	16.8 ± 2.1	13.7 ± 1.8	21.9 ± 2.8	17.7 ± 4.1	28.5 ± 6.5	22.3 ± 3.4	33.8 ± 2.1	<0.001
Fructose (g)	19.8 ± 1.7	10.5 ± 1.3	15.7 ± 1.6	13.6 ± 2.2	11.7 ± 1.2	20.5 ± 3.2	25.5 ± 4.8	23.3 ± 2.4	18.7 ± 1.1	0.001
Total Dietary Fiber (g)	22.0 ± 1.4	19.0 ± 1.5	21.5 ± 1.6	18.6 ± 1.4	17.1 ± 1.3	28.2 ± 4.7	23.6 ± 3.2	27.1 ± 2.9	19.4 ± 0.6	<0.001
Glycemic Index (%)	85.4 ± 1.4	80.3 ± 1.8	74.0 ± 1.5	79.8 ± 2.0	81.0 ± 1.3	80.9 ± 1.5	83.9 ± 1.9	83.9 ± 2.1	85.7 ± 0.5	<0.001
Glycemic Load (g)	158.9 ± 10.5	159.2 ± 20.3	122.8 ± 8.6	136.3 ± 9.8	118.6 ± 7.0	156.5 ± 18.4	180.2 ± 20.0	177.9 ± 14.9	181.4 ± 5.4	<0.001
Sodium (mg)	3115.8 ± 218.1	3663.6 ± 323.2	4313.4 ± 257.0	3779.4 ± 259.4	4134.4 ± 415.0	3936.0 ± 558.8	4075.5 ± 441.0	4509.4 ± 500.1	4047.7 ± 126.9	0.05
Water (ml)	1999.0 ± 90.8	2377.1 ± 138.6	2246.7 ± 103.0	2189.6 ± 121.1	2314.6 ± 93.0	2246.3 ± 236.5	2300.4 ± 159.4	3259.0 ± 242.4	2488.1 ± 59.4	<0.001

^a^ Result from statistical analysis using One-way ANOVA.

**Table 2:** Results from Principle Components Factor Analysis of Dietary Intakes from Adults with Type 2 Diabetes From Nine Countries.

USA (N = 316)					Argentina (N = 63)				
**Factor**	**1**	**2**	**3**	**4**	**Factor**	**1**	**2**	**3**	**4**
Eigenvalue	1.65	1.53	1.37	1.32	Eigenvalue	1.84	1.63	1.47	1.28
Variance (%)	11.79	10.93	9.78	9.43	Variance (%)	16.7	14.8	13.4	11.6
Fruits		0.54	0.23		Fruits	0.39	0.30		0.31
Vegetables		0.38			Vegetables	0.24	0.61	0.22	0.28
Grains, Breads, Starches, Cereals	0.38		0.57		Grains, Breads, Starches, Cereals	0.52			
Red Meats	0.43			0.36	Red Meats				0.85
White Meats and Fish		0.74			White Meats and Fish	0.79	0.21		
Legumes and Plant-Based Proteins				0.50	Legumes and Plant-Based Proteins				
Dairy			0.52		Dairy				
Desserts and Sweets	0.28			0.41	Desserts and Sweets			0.30	
Fats (Monounsaturated)			0.59		Fats (Monounsaturated)		0.77	0.26	
Fats (Polyunsaturated and Saturated)	0.73				Fats (Polyunsaturated and Saturated)	0.83			
Water			0.26		Water				0.25
Beverages (Noncaloric)	0.58				Beverages (Noncaloric)			0.64	
Beverages (Sweetened)					Beverages (Sweetened)				
Alcohol	0.52				Alcohol				
**Germany (N = 42)**					**Poland (N = 44)**				
**Factor**	**1**	**2**	**3**		**Factor**	**1**	**2**	**3**	
Eigenvalue	2.53	1.72	1.53		Eigenvalue	2.07	1.97	1.64	
Variance (%)	21.05	14.35	12.77		Variance (%)	18.8	17.9	14.9	
Fruits			0.63		Fruits	0.45	0.33		
Vegetables			0.21		Vegetables			0.71	
Grains, Breads, Starches, Cereals	0.90				Grains, Breads, Starches, Cereals		0.80		
Red Meat		0.23			Red Meats				
White Meats and Fish					White Meats and Fish	0.42	0.46	0.48	
Legumes and Plant-Based Proteins					Legumes and Plant-Based Proteins				
Dairy		0.60			Dairy		0.27		
Sweets	0.95				Desserts and Sweets		0.24	0.50	
Fats (Monounsaturated)		0.74			Fats (Monounsaturated)	0.71			
Fats (Polyunsaturated and Saturated)	0.88				Fats (Polyunsaturated and Saturated)		0.78	0.36	
Water			0.75		Water	0.54			
Beverages (Noncaloric)		0.64			Beverages (Noncaloric)	0.59			
Beverages (Sweetened)					Beverages (Sweetened)				
Alcohol					Alcohol				
**Serbia (N = 39)**					**Slovakia (N = 31)**				
**Factor**	**1**	**2**	**3**	**4**	**Factor**	**1**	**2**	**3**	**4**
Eigenvalue	1.75	1.58	1.52	1.49	Eigenvalue	2.04	1.90	1.77	1.60
Variance (%)	15.89	14.36	13.82	13.55	Variance (%)	17	15.9	14.8	13.4
Fruits		0.52			Fruits	0.78			
Vegetables	0.41		0.67		Vegetables	0.62			
Grains, Breads, Starches, Cereals		0.59		0.29	Grains, Breads, Starches, Cereals	0.32	0.59	0.32	
Red Meats			0.21	0.32	Red Meats				
White Meats and Fish				0.75	White Meats and Fish				0.93
Legumes and Plant-Based Proteins					Legumes and Plant-Based Proteins				
Dairy			0.80		Dairy	0.31	0.62		0.35
Desserts and Sweets	0.89				Desserts and Sweets	0.59		0.23	
Fats (Monounsaturated)				0.80	Fats (Monounsaturated)	0.20			
Fats (Polyunsaturated and Saturated)	0.72				Fats (Polyunsaturated and Saturated)			0.76	0.29
Water					Water			0.73	
Beverages (Noncaloric)		0.71	0.46		Beverages (Noncaloric)	0.49			0.25
Beverages (Sweetened)					Beverages (Sweetened)			0.82	
Alcohol					Alcohol				
**Slovenia (N = 22)**					**Spain (N = 27)**				
**Factor**	**1**	**2**	**3**	**4**	**Factor**	**1**	**2**	**3**	**4**
Eigenvalue	3.10	3.02	1.69	1.43	Eigenvalue	2.31	2.11	1.67	1.65
Variance (%)	23.83	23.21	12.99	9.53	Variance (%)	17.79	16.24	12.83	12.69
Fruits		0.22		0.72	Fruits		0.21	0.67	
Vegetables	0.75		0.31		Vegetables			0.74	
Grains, Breads, Starches, Cereals	0.93			0.56	Grains, Breads, Starches, Cereals	0.68	0.27		
Red Meats	0.82				Red Meats		0.38		0.70
White Meats and Fish				0.24	White Meats and Fish	0.60		0.25	
Legumes and Plant-Based Proteins	0.29	0.60	0.56		Legumes and Plant-Based Proteins				
Dairy	0.42	0.59		0.35	Dairy			0.36	
Desserts and Sweets		0.50	0.68		Desserts and Sweets	0.85			
Fats (Monounsaturated)	0.29		0.46		Fats (Monounsaturated)		0.77	0.29	
Fats (Polyunsaturated and Saturated)	0.69				Fats (Polyunsaturated and Saturated)		0.81		
Water	0.29	0.44			Water				
Beverages (Noncaloric)		0.72			Beverages (Noncaloric)	0.58			0.53
Beverages (Sweetened)					Beverages (Sweetened)	0.52	0.40		
Alcohol					Alcohol		0.23		
**Turkey (N = 31)**									
**Factor**	**1**	**2**	**3**						
Eigenvalue	2.85	2.04	1.85						
Variance (%)	23.78	17.03	15.44						
Fruits			0.61						
Vegetables	0.78								
Grains, Breads, Starches, Cereals	0.78								
Red Meats	0.87								
White Meats and Fish									
Legumes and Plant-Based Proteins			0.69						
Dairy	0.82								
Desserts and Sweets		0.28							
Fats (Monounsaturated)		0.94							
Fats (Polyunsaturated and Saturated)		0.87							
Water			0.20						
Beverages (Noncaloric)									
Beverages (Sweetened)									
Alcohol									

**Table 3:** Food Group Definitions of Dietary Pattern Labels Assigned to the Factors and Countries Identified by Factor.

Pattern Label	Most Frequent Food Groups	Country Factor			
		1	2	3	4
Mediterranean-like	Fruits, Grains, White Meats, Fish, Dairy, Monounsaturated Fats	Poland Slovakia	Argentina	Spain	Serbia Slovenia
Calorie Dense	Grains, Starches, Meats, Desserts, Sweets, Fats, Alcohol	Germany USA Spain	Turkey	Slovakia	
Vegetarian-Lacto	Fruits, Vegetables, Legumes, Dairy, Desserts, Sweets, Fats, Water	Serbia	Poland Slovenia	Argentina	
Vegan	Fruits, Vegetables, Grains, Legumes, Water		Serbia	Germany Slovenia Turkey	
Hearty	Vegetables, Breads, Starches, Red Meats, Dairy	Turkey	Germany	Serbia	
Varied	Vegetables, Grains, Breads, Starches, Red Meats, Legumes, Dairy, Fats, Water	Slovenia	Spain		
Vegetarian-Pesco	Fruits, Vegetables, Legumes, Fish, Desserts, Sweets, Fats	Argentina		Poland	
Low Carbohydrate	Fruits, Vegetables, Red or White Meats		USA		Argentina
High Fat	Red Meats, Legumes, Desserts, Polyunsaturated/Saturated Fats				Spain USA
Breakfast	Cereals, Grains, Breads, Dairy, Fats		Slovakia	USA	
Bland	White Meats, Fish, Dairy, Polyunsaturated/Saturated Fats				Slovakia

**Table 4:** Healthy Eating Index-2015 Component and Total Diet Quality Scores by Country.

	Score Range	Argentina	Germany	Poland	Serbia	Slovakia	Slovenia	Spain	Turkey	USA	*p* Value ^a^
Adequacy Components ^b^											
Total Fruits	0–5	2.56 ± 0.03	1.48 ± 0.04	2.18 ± 0.04	2.08 ± 0.04	2.19 ± 0.05	2.22 ± 0.08	3.00 ± 0.06	2.74 ± 0.06	1.69 ± 0.10	0.001
Whole Fruits	0–5	2.49 ± 0.04	1.71 ± 0.05	3.00 ± 0.05	2.67 ± 0.06	3.32 ± 0.06	3.11 ± 0.10	3.81 ± 0.06	3.55 ± 0.05	1.95 ± 0.12	0.001
Total Vegetables	0–5	3.22 ± 0.03	2.83 ± 0.04	4.14 ± 0.03	2.87 ± 0.04	3.52 ± 0.05	4.28 ± 0.06	2.67 ± 0.06	4.23 ± 0.04	2.95 ± 0.09	0.001
Greens and Beans	0–5	0.71 ± 0.03	1.29 ± 0.05	0.82 ± 0.04	0.74 ± 0.05	1.32 ± 0.07	2.89 ± 0.13	1.30 ± 0.08	2.68 ± 0.08	1.41 ± 0.12	0.001
Whole Grains	0–10	1.79 ± 0.05	2.21 ± 0.09	2.48 ± 0.09	2.03 ± 0.10	3.71 ± 0.14	3.56 ± 0.26	2.15 ± 0.14	2.77 ± 0.13	3.38 ± 0.23	0.14
Dairy	0–10	5.62 ± 0.06	4.71 ± 0.09	4.00 ± 0.08	6.67 ± 0.10	3.74 ± 0.10	5.00 ± 0.21	4.89 ± 0.12	6.39 ± 0.08	4.58 ± 0.19	0.001
Total Protein foods	0–5	4.00 ± 0.03	3.60 ± 0.04	4.64 ± 0.02	4.13 ± 0.04	4.71 ± 0.03	4.61 ± 0.05	4.33 ± 0.05	3.65 ± 0.06	4.52 ± 0.06	0.001
Seafood and Plant Proteins	0–5	0.16 ± 0.01	0.93 ± 0.04	1.70 ± 0.05	1.21 ± 0.05	1.84 ± 0.08	2.61 ± 0.13	2.11 ± 0.09	2.35 ± 0.07	2.33 ± 0.13	0.001
Fatty Acids ^c^	0–10	4.19 ± 0.06	2.71 ± 0.08	3.84 ± 0.08	3.97 ± 0.10	4.13 ± 0.11	6.06 ± 0.19	6.44 ± 0.15	4.61 ± 0.11	4.63 ± 0.20	0.005
Moderation Components ^d^											
Refined Grains	0–10	4.78 ± 0.07	5.81 ± 0.09	5.09 ± 0.09	4.72 ± 0.11	7.29 ± 0.11	7.61 ± 0.18	6.56 ± 0.13	5.06 ± 0.13	6.15 ± 0.21	0.003
Sodium	0–10	4.00 ± 0.06	5.07 ± 0.10	0.95 ± 0.06	2.05 ± 0.07	2.03 ± 0.10	2.17 ± 0.17	3.56 ± 0.13	1.74 ± 0.10	2.88 ± 0.17	0.001
Added Sugars	0–10	6.42 ± 0.19	7.63 ± 0.15	5.11 ± 0.06	5.08 ± 0.10	4.57 ± 0.04	4.94 ± 0.14	5.90 ± 0.12	5.95 ± 0.06	7.90 ± 0.20	0.001
Saturated Fats	0–10	6.38 ± 0.13	6.49 ± 0.12	6.70 ± 0.14	6.38 ± 0.05	5.33 ± 0.06	5.45 ± 0.09	6.21 ± 0.11	6.23 ± 0.07	6.97 ± 0.16	0.002
Total HEI Score	0–10	50.38 ± 0.21	47.93 ± 0.31	51.80 ± 0.24	51.87 ± 0.35	56.06 ± 0.33	63.78 ± 0.65	55.89 ± 0.05	58.35 ± 0.37	52.21 ± 0.04	0.001

^a^ Results from One-way ANOVA; ^b^ Higher score indicates better compliance with the Dietary Guidelines; ^c^ Based on ratio of unsaturated to saturated fats; ^d^ Lower score indicates better compliance with the Dietary Guidelines.

**Table 5:** Proportion of Subjects in Each Country with Poor Diet Quality Based on Healthy Eating Index-2015 (HEI-2015) Total Diet Quality Score.

	Poor Diet Quality	Needs Improvement	Good Diet Quality
	<51	51–80	>80
	(percentage obtaining score)		
Argentina	42.6	55.8	1.6
Germany	62.2	37.8	0.0
Poland	43.2	56.8	0.0
Serbia	41.7	55.5	2.8
Slovakia	26.7	73.3	0.0
Slovenia	11.8	82.3	5.9
Spain	32.0	64.0	4.0
Turkey	27.6	69.0	3.4
United States	42.5	56.4	1.1

## Supplementary Materials

The following are available online at https://www.mdpi.com/2072-6643/12/7/2027/s1, Table S1: The NDSR Output--Driven Food Groups (*n* = 14) and their Component Food and Beverage Items (*n* = 168) Used in Dietary Pattern Analysis. Figure S1: Results from Spearman Rank-Order Correlation Matrix with r ≥ 0.20 Confirming Positive Association Between Individual Food or Beverage Item and Primary Food Group.

## Abbreviations

BMI	body mass index
Core	Vanderbilt diet, body composition, and human metabolism core
EPIC	European prospective investigation of cancer
FPG	fasting plasma glucose
HbA1c	hemoglobin A1c
HEI	healthy eating index
HOMA-IR	homeostasis model of insulin resistance
IPAQ	international physical activity questionnaire
IRB	institutional review board
Kg	kilogram
M	meter
Mol	mole
Mmol	milli mole
MUFA	Monounsaturated fat
NDSR	nutrition data system for research
PCA	principal component analysis
PUFA	Polyunsaturated fat
SFA	Saturated fat
SPSS	statistical package for social sciences
T2DM	type 2 diabetes mellitus
