# Supplementary material for: Exploring the Diets of Adults with Obesity and Type II Diabetes from Nine Diverse Countries: Dietary Intakes, Patterns, and Quality
Source: Nutrients. 2020 Jul 8;12(7):2027. doi: 10.3390/nu12072027 (PMC7400897; doi:10.3390/nu12072027)
Supplement: Supplementary file 1 [file nutrients-12-02027-s001.pdf]

Supplemental Table S1. The NDSR Output--Driven Food Groups (n = 14) and their Component Food and Beverage Items (n = 168) Used in Dietary Pattern Analysis [a]

Food Group	Food and Beverage Items
Fruits	fruits, fruit juice, dried fruits, fried fruits
Vegetables	green vegetables, yellow vegetables, orange vegetables, tomato, fried vegetables, vegetable juice, pickled vegetables
Grains, Breads, Starches	whole grains, mixed grains, refined grains, pasta, rice, oatmeal, ready-to-eat cereals, loaf-type breads, rolls, quick breads, corn muffins, tortillas, crackers, snack bars, snack chips, corn, popcorn, potato
Red Meats	beef, veal, lamb, pork, game, cold cuts, sausage, organ meats, eggs
White Meats and Fish	poultry, fresh fish, smoked fish, fried fish, shellfish
Legumes and Plant-Based Proteins	legumes, beans, soy products, meat alternatives, egg substitutes
Dairy	milk, ready-to-drink flavored milk, milk beverage powder/dry milk, cheese, yogurt
Desserts and Sweets	frozen dairy and non-dairy desserts, puddings, cakes, cookies, pies, pastries, doughnuts, cobblers, sugar, syrups, honey, jam, jelly, preserves, sweet sauces, chocolate candy, non-chocolate candy, frosting, glaze, miscellaneous desserts
Fats (Monounsaturated)	avocado, nuts, seeds, nut and seed butters, olive oil
Fats (Polyunsaturated and Saturated)	margarine, shortening, butter, salad dressings, gravy, sauces, cream
Water	tap water, bottled water (plain), plain coffee, plain tea
Beverages (Unsweetened and Artificially Sweetened)	artificially sweetened soft drinks, artificially sweetened fruit drinks, artificially sweetened tea, artificially sweetened coffee, artificially sweetened coffee substitutes, artificially sweetened water, soup broths, non-alcoholic light beer
Beverages (Sweetened)	sweetened soft drinks, sweetened fruit drinks, sweetened tea, sweetened coffee, sweetened coffee substitutes, sweetened water, non-alcoholic beer
Alcohol	Beer, ale, wine, distilled liquor, cordials, liqueurs

[a] NDSR Serving Count Totals File: 168 food and beverage items were combined into 14 primary food groups based on ingredients, energy, and macronutrient composition within a standard serving size.

USA								Food Group						
**Food Item**	Fruits	Vegetables	Starches	Red Meats	White Meats and Fish	Legumes and Plant Proteins	Dairy	Desserts and Sweets	Fats MUFA	Fats PUFA SFA	Water	Bev. Unsweet	Bev. Sweet	Alcohol
Citrus Juice	0.32													
Fruit Juice excluding Citrus	0.32													
Citrus Fruit	0.41													
Fruit excluding Citrus	0.77													
Dark-Green Vegetables		0.41												
Deep-Yellow Vegetables		0.39												
Tomato		0.38				0.23								
Other Vegetables		0.77												
Vegetable Juice		0.17												
Pickled Foods		0.24												
Refined Grains			0.20			0.26			0.31					
Refined Grain Loaf Breads and Rolls			0.41											
Refined Grain Quick Breads, Muffins, Tortillas			0.22							0.20				
Pasta			0.27											
Beef				0.59										
Cold Cuts and Sausage				0.39										
Lean Cold Cuts and Sausage				0.30										
Eggs				0.39						0.26				
Fresh Pork					0.22									
Cured Pork					0.21									
Lean Cured Pork					0.21									
Poultry					0.28									
Lean Poultry					0.36									
Fried Chicken					0.25									
Lean Fish					0.30									
Legumes						0.91								
Meat Alternatives						0.40								
Milk - Reduced Fat							0.40							
Milk - Low Fat and Fat Free							0.23							
Cheese - Full Fat				0.20			0.52							
Cheese - Reduced Fat							0.35							
Sugar								0.28						
Sugar Substitute								0.64				0.22		
Chocolate Candy								0.20						
Other Candy								0.20						
Cakes, Cookies, Pies, Pastries, Danish, Doughnuts, Cobblers								0.35		0.20				
Frozen Dairy Dessert								0.27						
Avocado						0.20								
Nuts and Seeds									0.50					
Nut and Seed Butters									0.23					
Oil								0.22	0.65					
Cream - Nondairy										0.22				
Margarine - Reduced Fat										0.30				
Shortening					0.20					0.29				
Butter - Regular										0.42				
Salad Dressing - Regular		0.23								0.37				
Salad Dressing - Reduced Fat		0.25												
Gravy and Sauces - Regular										0.20				
Gravy and Sauces - Reduced Fat				0.25						0.39				
Soup Broth											0.20			
Tea												0.40		
Tea - Sweetened													0.25	
Coffee								0.27				0.47		
Soft Drinks - Sweetened													0.76	
Soft Drinks - Artificially Sweetened												0.53		
Fruit Drinks - Sweetened													0.51	
Fruit Drinks - Artificially Sweetened												0.23		
Beer and Ales														0.56
Liquor														0.47
Wine														0.68

Argentina	Food Group											
Food Item	Fruits	Vegetables	Starches	Red Meats	White Meats and Fish	Dairy	Desserts and Sweets	Fats MUFA	Fats PUFA SFA	Bev. Unsweet	Bev. Sweet	Alcohol
Citrus Juice	0.60											
Citrus Fruit	0.43											
Fruit excluding Citrus	0.48				0.27							
Dark-green Vegetables		0.30										
Deep-yellow Vegetables		0.52										
Tomato		0.44						0.27				
Other Vegetables		0.79						0.27				
Pickled Foods												0.43
White Potatoes			0.26									
Starchy Vegetables		0.29										
Refined Grains			0.34		0.27							
Refined Grain Loaf Breads and Rolls			0.66									
Refined Grain Quick Breads, Muffins, Tortillas												0.25
Refined Grain Crackers		0.34										
Whole Grain Cereals					0.29							
Beef				0.93								
Game												0.43
Cold Cuts and Sausage												0.29
Lean Cold Cuts and Sausage												0.46
Lean Fresh Pork					0.25				0.27			
Lean Cured Pork									0.29			
Poultry					0.55							
Lean Poultry					0.49							
Milk - Reduced Fat						0.34	0.26					
Milk - Low Fat and Fat Free						0.27						
Cheese - Full Fat						0.65						
Cheese - Reduced Fat	0.26	0.25										
Yogurt - Low Fat						0.25						
Yogurt - Artificially Sweetened Low Fat						0.26						
Cakes, Cookies, Pies, Pastries, Danish, Doughnuts, Cobblers							0.26					
Frozen Dairy Dessert												0.28
Sugar Substitute			0.32				0.74					
Syrup, Honey, Jam, Jelly, Preserves							0.35					
Chocolate Candy											0.31	
Oil		0.29						1.00				
Margarine - Reduced Fat									0.53			
Shortening	0.27				0.31				0.51			
Butter - Regular							0.29		0.45			
Salad Dressing - Regular									0.53			
Gravy and Sauces - Reduced Fat			0.28		0.28							
Tea										0.63	0.28	
Soft Drinks - Sweetened											0.54	
Soft Drinks - Artificially Sweetened										0.46		
Fruit Drinks - Sweetened											0.71	
Water - Sweetened									0.25		0.43	0.30
Beer and Ales												0.63
Wine				0.29							0.25	0.75

Germany	Food Group													
Food Item	Fruits	Vegetables	Starches	Red Meats	White Meats and Fish	Legumes and Plant Proteins	Dairy	Desserts and Sweets	Fats MUFA	Fats PUFA SFA	Water	Bev. Unsweet	Bev. Sweet	Alcohol
Citrus Juice	0.49													
Citrus Fruit	0.52													
Fruit excluding Citrus	0.53													
Dark-green Vegetables		0.43												
Tomato		0.47												
Other Vegetables		0.72												
Pickled Foods										0.35				
Fried Potatoes			0.32											
Whole Grain Loaf Breads and Rolls			0.40											
Refined Grain Loaf Breads and Rolls			0.49											
Refined Grain Quick Breads, Muffins, Tortillas														0.34
Whole Grain Crackers			0.31						0.41					
Pasta									0.31					
Whole Grain Cereals				0.35										0.31
Refined Grain Snack Chips							0.31							
Beef				0.51										
Lean Beef														0.31
Cold Cuts and Sausage				0.51										
Fresh Pork					0.56									
Cured Pork					0.35									
Lean Poultry					0.39									
Legumes						0.88								
Cheese - Full Fat							0.60							
Cheese - Low Fat and Fat Free							0.32							
Yogurt - Low Fat							0.33							
Cakes, Cookies, Pies, Pastries, Danish, Doughnuts, Cobblers										0.31				
Sugar								0.48						
Sugar Substitute			0.34					0.61						
Syrup, Honey, Jam, Jelly, Preserves			0.33					0.47						
Candy, not chocolate								0.35						
Nuts and Seeds									0.42					
Oil									0.90					
Cream - Nondairy								0.31						
Shortening					0.39									
Butter - Regular						0.49				0.67				0.32
Salad Dressing - Regular		0.39												0.31
Gravy and Sauces - Regular		0.31												
Soup Broth											0.42			
Soft Drinks - Artificially Sweetened												0.56		
Tea												0.40		
Coffee												0.55		
Beer and Ales														0.37
Wine														0.87

**Poland**	**Food Group**											
**Food Item**	Fruits	Vegetables	Starches	Red Meats	White Meats and Fish	Legumes and Plant Proteins	Dairy	Desserts and Sweets	Fats MUFA	Fats PUFA SFA	Bev. Unsweet	Bev. Sweet
Citrus Juice												
Citrus Fruit	0.48	0.32										
Fruit excluding Citrus	0.72											
Dark-green Vegetables		0.31										
Other Vegetables		0.58						0.39	0.34			0.39
Vegetable Juice		0.39										
Pickled Foods		0.64										
White Potatoes	0.46		0.44									
Whole Grain Loaf Breads and Rolls			0.36			0.32						
Mixed Grain Loaf Breads and Rolls												
Refined Grain Loaf Breads and Rolls			0.51									0.36
Whole Grain Crackers				0.33								
Beef				0.55								
Cold Cuts and Sausage				0.56								
Lean Cold Cuts and Sausage				0.36								
Fresh Pork					0.35	0.32						
Cured Pork			0.31		0.45							
Lean Cured Pork						0.56						
Poultry					0.30							
Fish					0.35							
Lean Fish									0.30			
Legumes				0.31		1.00						
Milk - Low Fat and Fat Free							0.38					
Cheese - Full Fat							0.54					
Cheese - Reduced Fat							0.51					
Yogurt - Low Fat							0.35					
Yogurt - Fat Free						0.54						
Cakes, Cookies, Pies, Pastries, Danish, Doughnuts, Cobblers								0.45				
Sugar								0.69				
Sugar Substitute								0.37				0.48
Syrup, Honey, Jam, Jelly, Preserves								0.39				
Oil		0.30							1.00			
Cream												0.49
Margarine - Regular												
Margarine - Reduced Fat						0.51						
Shortening					0.35							
Butter - Regular										0.72		
Salad Dressing - Regular								0.40				
Gravy and Sauces - Reduced Fat										0.53		
Tea											0.87	
Tea - Sweetened												0.38
Soft Drinks - Sweetened								0.36				0.76
Fruit Drinks - Sweetened												0.46

**Serbia**	**Food Group**												
**Food Item**	Fruits	Vegetables	Starches	Red Meats	White Meats and Fish	Legumes and Plant Proteins	Dairy	Desserts and Sweets	Fats MUFA	Fats PUFA SFA	Bev. Unsweet	Bev. Sweet	Alcohol
Citrus Juice	0.36												
Citrus Fruit	0.63												
Fruit excluding Citrus	0.62												
Deep-yellow Vegetables				0.32									
Other Vegetables		0.52		0.43			0.44						0.32
Pickled Foods		0.53											
Starchy Vegetables			0.32								0.38		0.42
Refined Grains							0.34			0.41			
Refined Grain Loaf Breads and Rolls			0.75										
Mixed Grain Cereals	0.35												0.47
Beef				0.38									
Lamb				0.35									
Game													0.72
Cold Cuts and Sausage				0.60									
Fried Chicken						0.47							
Fish												0.66	
Lean Fish									0.37				
Legumes						1.00		0.34					
Milk - Whole													0.50
Milk - Reduced Fat							0.39						
Milk - Low Fat and Fat Free											0.34		0.45
Cheese - Full Fat							0.73			0.34			
Cheese - Reduced Fat					0.32								
Yogurt - Low Fat	0.33												
Cakes, Cookies, Pies, Pastries, Danish, Doughnuts, Cobblers								0.58				0.35	
Pudding and Other Dairy Dessert													0.68
Sugar						0.51		0.69					
Sugar Substitute		0.39						0.34					0.36
Syrup, Honey, Jam, Jelly, Preserves								0.33	0.32				
Nuts and Seeds									0.37				
Oil		0.35		0.41					0.91				
Cream			0.34							0.51			
Margarine - Reduced Fat										0.61			
Shortening						0.34							
Butter - Regular										0.45			
Salad Dressing - Regular									0.32			0.34	
Gravy and Sauces - Reduced Fat				0.45						0.53			
Tea											0.67		
Coffee		0.35									0.71		
Fruit Drinks - Artificially Sweetened						0.52							
Fruit Drinks - Sweetened												0.56	
Soft Drinks - Sweetened												0.81	
Wine													1.00

Slovakia	Food Group											
**Food Item**	Fruits	Vegetables	Starches	Red Meats	White Meats and Fish	Legumes and Plant Proteins	Dairy	Desserts and Sweets	Fats MUFA	Fats PUFA SFA	Bev. Unsweet	Alcohol
Citrus Fruit	0.75							0.38				
Fruit excluding Citrus	0.45											
Vegetables		0.48				0.47						
White Potatoes		0.50				0.37						
Pasta											0.43	
Lean Beef												
Cold Cuts and Sausage				0.65								
Fresh Pork					0.52						0.37	
Lean Fish												0.55
Legumes		0.36				1.00						
Meat Alternatives						0.48						
Milk - Reduced Fat						0.41						
Cheese - Full Fat			0.38				0.47					
Yogurt - Low Fat											0.38	
Mixed Grain Cakes, Cookies, Pies, Pastries, Danish, Doughnuts, Cobblers								0.36				
Refined Grain Cakes, Cookies, Pies, Pastries, Danish, Doughnuts, Cobblers			0.36									
Sugar								0.82	0.47			
Sugar Substitute								0.55				
Nuts and Seeds									0.43			
Oil								0.43	0.92			
Shortening					0.40							0.36
Butter - Regular										0.53		
Gravy and Sauces - Reduced Fat								0.47				
Tea											0.81	
Coffee	0.36											
Beer and Ales												0.48
Wine												0.84

Slovenia	Food Group												
**Food Item**	Fruits	Vegetables	Starches	Red Meats	White Meats and Fish	Legumes and Plant Proteins	Dairy	Desserts and Sweets	Fats MUFA	Fats PUFA SFA	Bev. Unsweet	Bev. Sweet	Alcohol
Citrus Fruit	0.55												
Fruit excluding Citrus	0.87												
Dark-green Vegetables		0.58											
Deep-yellow Vegetables		0.65											
Other Vegetables					0.48								0.54
Pickled Foods		0.56											
White Potatoes			0.48										
Whole Grain Loaf Breads and Rolls			0.53										
Mixed Grain Loaf Breads and Rolls			0.64										
Refined Grain Loaf Breads and Rolls				0.54									
Cold Cuts and Sausage				0.65									
Fresh Pork		0.66										0.56	
Poultry					0.48								
Lean Poultry													0.51
Legumes						0.96							
Cheese - Reduced Fat							0.60						
Yogurt - Low Fat	0.58												
Mixed Grain Cakes, Cookies, Pies, Pastries, Danish, Doughnuts, Cobblers	0.55												
Sugar								0.43					
Sugar Substitute								0.51					
Chocolate Candy												0.73	
Nuts and Seeds										0.59			
Oil									0.90				
Margarine - Reduced Fat										0.60			
Butter - Regular									0.52			0.65	
Tea										0.60	0.63		
Coffee - Sweetened												1.00	
Wine					0.83								0.86

**Spain**	**Food Group**											
**Food Item**	Fruits	Vegetables	Starches	Red Meats	White Meats and Fish	Dairy	Desserts and Sweets	Fats MUFA	Fats PUFA SFA	Bev. Unsweet	Bev. Sweet	Alcohol
Citrus Fruit	0.45											
Fruit excluding Citrus	0.68											
Tomato		0.55										
Other Vegetables		0.90										
Pickled Foods		0.45										
Fried Potatoes			0.50									
Refned Grains									0.55			
Whole Grain Loaf Breads and Rolls			0.41									
Refined Grain Loaf Breads and Rolls			0.54	0.52								
Whole Grain Cereals												0.45
Beef				0.49								
Cold Cuts and Sausage			0.47	0.44							0.40	
Eggs				0.57				0.52	0.66		0.41	
Cured Pork					0.44							
Poultry					0.51							0.54
Milk - Low Fat and Fat Free						0.47						
Cheese - Full Fat						0.79						
Yogurt - Fat Free			0.43				0.50			0.51		
Sugar Substitute							0.82					
Nuts and Seeds												0.39
Oil								0.94	0.41			
Margarine - Regular	0.38											
Butter - Regular								0.51	0.47			
Salad Dressing - Regular									0.58			
Tea										0.48		
Coffee										0.64		
Soft Drinks - Sweetened			0.40	0.40							0.89	
Soft Drinks - Artificially Sweetened										0.44		
Distilled Liquor												0.45
Wine					0.42							0.87

**Turkey**	**Food Group**										
**Food Item**	Fruits	Vegetables	Starches	Red Meats	White Meats and Fish	Legumes and Plant Proteins	Dairy	Desserts and Sweets	Fats MUFA	Fats PUFA SFA	Bev. Unsweet
Citrus Juice				0.42							
Citrus Fruit	0.81										
Fruit excluding Citrus Fruit	0.78										
Dark-green Vegetables	0.37	0.36					0.37				
Tomato		0.58		0.49							
Other Vegetables		0.80		0.44							
Whole Grains	0.36										
Refined Grains			0.58				0.39				
Mixed Grain Loaf Breads and Rolls											
Refined Grain Loaf Breads and Rolls			0.39		0.37					0.51	0.42
White Potatoes										0.41	
Beef		0.49		0.80							
Lean Beef			0.39								
Organ Meats								0.41			
Eggs				0.39							
Poultry					0.49						
Lean Poultry										0.37	
Lean Fish					0.71						
Legumes	0.37					1.00					
Milk - Reduced Fat											0.51
Cheese - Full Fat							0.44				
Yogurt - Full Fat							0.47				
Cakes, Cookies, Pies, Pastries, Danish, Doughnuts, Cobblers								0.52			
Sugar				0.52							0.39
Other Candy								0.43			
Nuts and Seeds									0.51		
Oil									0.81		
Shortening					0.57					0.47	0.47
Butter - Regular					0.52					0.84	
Tea										0.36	0.99

Supplemental Figure S1: Results from Spearman Rank-Order Correlation Matrix with r ≥ 0.20 Confirming Positive Association Between Individual Food or Beverage Item and Primary Food Group.
